# Evaluation of CT Angiography Image Quality Acquired with Single-Energy Metal Artifact Reduction (SEMAR) Algorithm in Patients After Complex Endovascular Aortic Repair

**DOI:** 10.1007/s00270-017-1812-0

**Published:** 2017-10-30

**Authors:** M. A. A. D. Ragusi, R. W. van der Meer, R. M. S. Joemai, J. van Schaik, C. S. P. van Rijswijk

**Affiliations:** 1 Department of Radiology, Leiden University Medical Center, Albinusdreef 2, P.O. Box 9600, 2300RC Leiden, The Netherlands; 2 Department of Surgery, Leiden University Medical Center, Albinusdreef 2, P.O. Box 9600, 2300RC Leiden, The Netherlands

**Keywords:** Metal artifact reduction, SEMAR, Image quality, Computed tomography, Endovascular aortic repair

## Abstract

**Purpose:**

To evaluate the value of single-energy metal artifact reduction (SEMAR) algorithm on image quality in patients after complex endovascular aortic repair (EVAR) with fenestrated and branched devices.

**Methods:**

Routine follow-up computed tomography angiography (CTA) examinations were performed between February 2016 and May 2017 in 18 patients who underwent a complex EVAR procedure at our institution. Objective analysis was performed by measuring the standard deviation (SD) of attenuation (Hounsfield Units), and the contrast-to-noise ratio (CNR) in regions of interests in the stented visceral arteries. Subjective analysis of the degree of artifacts and stent visualization was performed independently by two interventional radiologists, blinded to the image reconstruction.

**Results:**

The SD of attenuation was significantly lower in all target visceral arteries (*p* < .001), the celiac artery (*p* = .002), the superior mesenteric artery (SMA; *p* = .043), and renal arteries (*p* < .001) in the CT images with SEMAR reconstruction. The CNR significantly increased in all SEMAR-reconstructed target visceral arteries (overall: *p* < .001, celiac artery: *p* = .009; SMA: *p* = .003; renal arteries: *p* < .001). The reviewers rated a significantly lower artifact degree in all target vessels (overall: *p* < .001, celiac artery: *p* = .001; SMA: *p* = .008; renal arteries: *p* < .001) and a significantly improved visualization of the stent patency in all target vessels (overall: *p* < .001, celiac artery: *p* = .031; SMA: *p* = .047; renal arteries: *p* < .001) in the SEMAR images. Overall preference of both reviewers was in favor of the SEMAR reconstruction in 15/18 cases (83%).

**Conclusion:**

Reconstruction with SEMAR algorithm significantly improves CTA image quality in patients after complex EVAR.

**Level of Evidence:**

Level 4, Case series.

## Introduction

Endovascular abdominal aortic aneurysm repair (EVAR) provides a safe and feasible alternative to traditional open surgical techniques [1–3]. Fenestrated and branched devices have been designed to incorporate visceral artery segments, thereby expanding the patient population suitable for endovascular repair. The design of the devices with scallops, fenestrations, or branches depends on the morphology of the aneurysm. Fenestrations and branches are connected to the visceral arteries with peripheral stents for fixation and sealing [4].

Computed tomography angiography (CTA) is currently the standard for long-term EVAR surveillance [5] to detect endoleaks, to evaluate stent patency, and to detect stent migration or disconnection [2–4, 6].

**Fig. 1:**
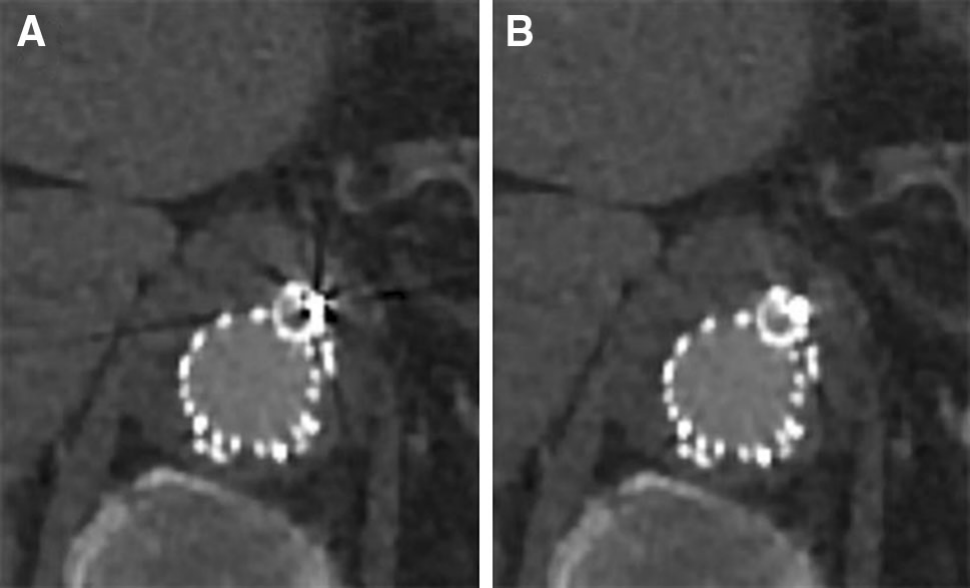
CT images (**A** standard (AIDR 3DE),** B** SEMAR) of a branch connected to the celiac artery in a 65-year-old male. Artifacts (black streaks) are reduced on the CT image with the SEMAR reconstruction compared to the standard reconstruction. All CT images are displayed with a window level of 300 HU and a window width of 1000 HU

**Fig. 2:**
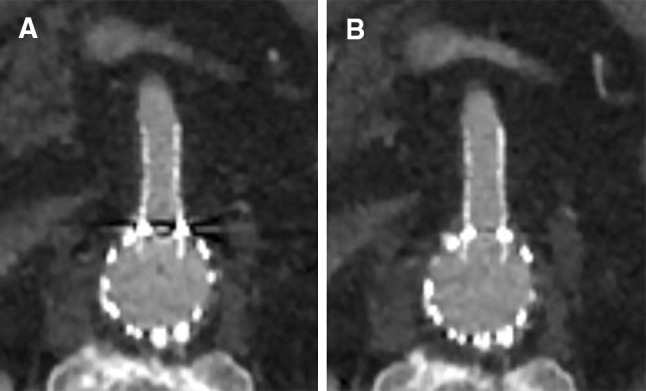
CT images (**A** standard (AIRD 3DE),** B** SEMAR) of a superior mesenteric artery in an 80-year-old male with a peripheral covered balloon-expendable stent in the fenestration. Almost complete reduction of artifacts in the SEMAR image

The metallic design of the devices and the radiopaque markers on the fenestrations, branches, and peripheral stents cause artifacts due to beam hardening and scatter that decrease image quality (Figs. 1, 2) [7–9]. These artifacts hamper visualization of the stent patency and reduce diagnostic confidence. Metal artifact reduction (MAR) tools might be helpful by increasing image quality and reducing the need for other diagnostic imaging tools such as duplex ultrasonography or digital subtraction angiography to diagnose possible complications.

Several MAR tools have been developed for use in clinical practice, including: dual-energy computed tomography (DECT) and MAR algorithms [10]. DECT is a scanning technique that obtains data at two different energy settings (typically 80 and 135 or 140 kVp). The monochromatic reconstructions at higher energy levels decrease the beam hardening artifacts by eliminating the lower energy quanta. Additional MAR algorithms can be used to further decrease metal artifacts. DECT-imaging might complicate the acquisition protocol and increase radiation dose [11].

Single-energy metal artifact reduction (SEMAR) algorithm is a CT MAR algorithm that can be used to reduce metal artifacts in both single-energy and dual-energy CT images [12, 13]. SEMAR corrects the metal artifacts based on a metal implanting technique with forward projections in the raw-data. Since “conventional” CT scanners acquire images at single-energy settings no adjustments of the acquisition protocol are required and there is no increased radiation dose.

Various authors have proved the positive impact of SEMAR on metal artifact reduction in CT images of patients with orthopedic prostheses [14, 15], embolization coils [8, 9, 15], and dental prostheses [13]. The impact of the SEMAR algorithm on CTA metal artifact reduction in patients after fenestrated and/or branched EVAR (F/B-EVAR) has not yet been investigated.

The purpose of our study was to evaluate the value of SEMAR algorithm on the image quality in single-energy images of patients after complex endovascular aortic repair with fenestrated and branched devices.

## Materials and Methods

### Patient Population

Between February 2016 and May 2017, we included patients with a routine CT follow-up examination after an F/B-EVAR procedure at our institution. All patients were included only once, and repeated CT examinations were excluded. Our follow-up CT protocol consists of a CT examination (without contrast, arterial, and delayed phase) within 30 days after treatment, followed by a CT examination annually. For this retrospective study, formal informed consent was not deemed necessary by the medical ethical board.

### Fenestrated/Branched-EVAR Devices and Peripheral Stents

Balloon-expandable Atrium Advanta stents (stainless steel, no radiopaque markers; Maquet, Rastatt, Germany) were deployed in all fenestrations to secure fixation. All branches were connected to the target vessels with Fluency stents (nitinol, tantalum markers; Bard, Tempe, AZ, USA) and fixated with a balloon-expandable Palmaz Genesis stent (stainless steel, no radiopaque markers; Cordis, Baar, Switzerland) in the branch. Self-expandable Cordis S.M.A.R.T. stents (nitinol, tantalum markers; Cordis, Baar, Switzerland) were used for relining. Relining was performed on indication to prevent kinking of the transition between the Fluency or Atrium Advanta stent and the target vessel to ensure a smooth alignment of the vessel with the stent.

### Acquisition Protocol

All patients were scanned on a 320-detector row CT scanner (Aquillion ONE Genesis edition, Toshiba Medical Systems, Otawara, Japan). The acquisition parameters for the abdominal CTA were as follows: 0.5 s rotation time, 120 kVp, 80-325 mAs (automated exposure control), slice thickness 0.5 mm, collimation of 80 × 0.5 mm, pitch of 65, and the matrix was 512 × 512. The amount of contrast (Ultravist 370; Bayer, Leverkusen, Germany) injected was based on the weight of the patient (1.3 ml/kg) and was injected over a period of 25 s followed by the injection of saline (0.5 ml/kg) over a period of 10 s. Two volumes with 1.0 mm slices were reconstructed for each patient, one using the standard reconstruction technique (Adaptive Iterative Dose Reduction 3D Enhanced (AIDR 3DE), Toshiba Medical Systems), further mentioned as standard CT, and one using the SEMAR algorithm (SEMAR, Toshiba Medical Systems). Reconstruction time of the SEMAR CT images was on average 30 min.

### Single-Energy Metal Artifact Reduction Algorithm

The first step in the SEMAR algorithm is to reconstruct the first-pass image through standard reconstruction and to automatically identify metal traces in this image by means of an HU threshold. The metal segment is identified in the original sinogram through forward projection and removed by linear interpolation using neighboring nonmetal measurements. The interpolated sinogram is reconstructed to create the second-pass image; this image is classified into several tissues (air, water, and bone) through a segmentation process. The second-pass image is forward projected onto the original metal trace sinogram to provide an estimation of the projection attributable to it. The original sinogram is then blended with the tissue-classified image sinogram. The third-pass image is created through a reconstruction of the blended sinogram in which the metal is added to the blended projection to obtain the final image [12].

### Objective Assessment of Image Quality

**Fig. 3:**
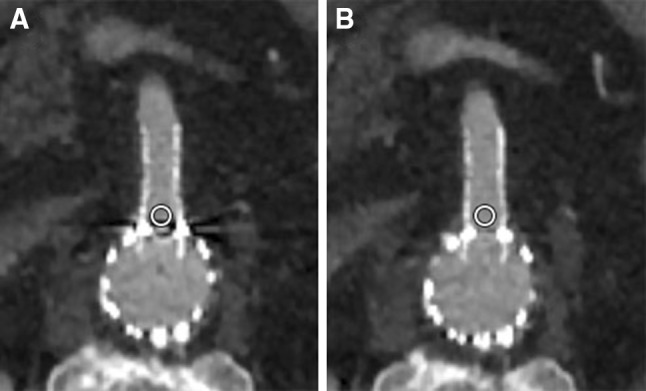
Example of ROI placement (**A** standard (AIDR 3DE), **B** SEMAR) proximal in the fenestration, with a balloon-expandable Atrium Advanta stent, in the superior mesenteric artery of an 80-year-old male

To objectively evaluate image quality, the images were exported to Philips IntelliSpace Portal (ISP) (Philips Healthcare, Eindhoven, The Netherlands), which is integrated into our PACS (IDS7, Sectra, Linköping, Sweden). Manually drawn circular regions of interest (ROI) were placed in the lumen of peripheral stents in the visceral arteries (celiac, superior mesenteric, and renal arteries) to measure the Hounsfield Units (HU; attenuation) and standard deviation of the HU in the ROI (noise [16]). These ROI were placed in the standard CT images and subsequently copied to the SEMAR CT images in exactly the same location. The ROI were manually placed proximally in the stents of the visceral arteries to include the most severe artifacts (Fig. 3). The inclusion of stent material or calcifications in the ROI was carefully avoided. The average ROI size was 10 mm^2^ for the celiac and superior mesenteric artery (SMA) and 6 mm^2^ for the renal arteries. ROI were drawn in air and in erector spinae muscle to determine the contrast-to-noise ratio [(attenuation of the vessel—attenuation of muscle)/SD air] [7]. CNR is a dimensionless number, it is used as a measure to compare image quality.

### Subjective Assessment of Image Quality

To evaluate the subjective image quality, two interventional radiologists (CR: 10 years of experience; RM: 5 years of experience), blinded with regard to the image reconstruction and patient information, independently evaluated the peripheral stents between the standard and SEMAR CT images in the arterial phase presented side by side. The following parameters were assessed: total degree of artifacts 1 = no artifacts; 2 = mild artifacts; 3 = moderate artifacts; 4 = strong artifacts; and 5 = extensive artifacts; and the visualization of the stent lumen 1 = exceptional visualization of the lumen; 2 = good visualization of the lumen; 3 = moderate visualization of the lumen; 4 = poor visualization of the lumen; and 5 = no visualization of the lumen. Finally, the radiologists indicated their preference for either the left or the right CT image.

### Statistics

Statistical analysis was performed with SPSS (version 23.0.0.2, IBM, Armonk, NY, USA). Variables are expressed as median (interquartile range) unless stated otherwise. The quantitative measurements between the SEMAR and the non-SEMAR images and the subjective analysis scale ratings were compared with the Wilcoxon signed-rank test. To assess inter-reader agreement, a linearly weighted *k* was calculated. A *p* value of less than 0.05 was considered to represent significant differences.

## Results

### Patients

A total of 18 follow-up CT examinations were included with the standard and SEMAR reconstructions. Mean patient age was 71.7 ± 5.4 years: range 64–81; 5 women and 13 men; BMI 26.2 ± 4.2. Twelve patients received a custom-made Cook Zenith endovascular graft (stainless steel; Cook Medical, Limerick, Ireland), and six patients received an off-the-shelf Cook Zenith t-branch endovascular graft (stainless steel; Cook Medical, Limerick, Ireland). Patient and stent characteristics are summarized in Table 1. Twelve patients were scanned within 30 days of the F/B-EVAR procedure, and 6 patients had a follow-up interval ranging between 2 months and 2.5 years after treatment.

**Table 1:** Patient and stent characteristics

Patient characteristics (*n* = 18)		
Age (mean ± SD)		71.7 ± 5.4
Women		5 (28%)
BMI (mean ± SD)		26.2 ± 4.2
Stent-type:	Custom-made	12 (67%)
	T-branch	6 (33%)
Stent characteristics for each visceral artery		
Celiac artery	# Included	12
	Fenestrations	2
	Branches	10
Stent diameter range (mm)		8–10
Superior mesenteric artery	# Included	16
	Fenestrations	6
	Branches	10
Stent diameter range (mm)		8–12
Renal artery	# Included	33
	Fenestrations	18
	Branches	15
Stent diameter range (mm)		5–8

In total, 61 branches and fenestrations were included in the evaluation. Three renal arteries were excluded in the objective analysis as sufficient ROI size could not be attained in two renal arteries, and one renal artery was completely occluded.

### Quantitative Assessment of Image Quality

In the objective analysis, the SEMAR reconstruction showed significantly reduced SD of the attenuation (HU) in the ROI in all target vessels (SEMAR: 38.3 (30.0) vs Standard: 62.8 (52.1), *p* < .001), the celiac artery (44.8 (35.2) vs 78.4 (80.6), *p* = .002), the SMA (40.1 (27.1) vs 56.9 (55.8), *p* = .043), and the renal arteries (34.2 (33.0) vs 60.8 (39.5), *p* < .001) (Table 2).

**Table 2:** Objective analysis of image quality of the Standard (AIDR 3De) and SEMAR reconstruction

	SD of attenuation (HU)			CNR		
	Standard	SEMAR	*p*	Standard	SEMAR	*p*
All visceral arteries (*n* = 58)	62.8 (52.1)	38.3 (30.0)	< .001	18.6 (9.7)	23.4 (8.3)	< .001
Celiac artery (*n* = 12)	78.4 (80.6)	44.8 (35.2)	.002	19.7 (13.4)	24.3 (9.3)	.009
Superior mesenteric artery (*n* = 16)	56.9 (55.8)	40.1 (27.1)	.043	19.4 (7.4)	23.9 (9.9)	.003
Renal arteries (*n* = 30)	60.8 (39.5)	34.2 (33.0)	< .001	17.8 (10.8)	22.4 (7.0)	< .001

The CNR was significantly higher in all target vessels in the SEMAR reconstructions compared to the standard reconstruction (overall: SEMAR: 23.4 (8.3) vs Standard: 18.6 (9.7), *p* < .001; celiac artery: 24.3 (9.3) vs 19.7 (13.4), *p* = .009; SMA: 23.9 (9.9) vs 19.4 (7.4), *p* = .003; and renal arteries: 22.4 (7.0) vs 17.8 (10.8), *p* < .001) (Table 2).

### Subjective Assessment of Image Quality

The reviewers rated a significantly lower degree of artifacts in the SEMAR reconstruction compared to the standard reconstruction in all target vessels (overall: SEMAR: 3 (1.5) vs Standard: 4 (1.5), *p* < .001; celiac artery: 2.5 (.5) vs 3.5 (.5), *p* = .001; SMA: 3 (1.9) vs 4 (1.3), *p* = .008; renal arteries: 3 (1.8) vs 4 (1.5), *p* < .001) (Table 3).

**Table 3:** Subjective analysis of degree of artifacts and lumen visualization of the Standard (AIDR 3De) en SEMAR reconstruction

	Artifact degree score			Visualization score		
	Standard	SEMAR	*p*	Standard	SEMAR	*p*
All visceral arteries (*n* = 61)	4 (1.5)	3 (1.5)	< .001	3 (1.5)	2.5 (1.5)	< .001
Celiac artery (*n* = 12)	3.5 (.5)	2.5 (.5)	.001	3 (1.5)	2.5 (1.8)	.031
Superior mesenteric artery (*n* = 16)	4 (1.3)	3 (1.9)	.008	3 (1.4)	2.5 (1.4)	.047
Renal arteries (*n* = 33)	4 (1.5)	3 (1.8)	< .001	4 (2.3)	3 (1.5)	< .001

Rating scale of the artifact degree: 1 = no artifacts; 2 = mild artifacts; 3 = moderate artifacts; 4 = strong artifacts; and 5 = extensive artifacts

Rating scale of the visualization: 1 = exceptional visualization of the lumen; 2 = good visualization of the lumen; 3 = moderate visualization of the lumen; 4 = poor visualization of the lumen; and 5 = no visualization of the lumen

Both reviewers rated an improved visualization of the lumen in the SEMAR reconstruction compared to the standard reconstruction in all target vessels (overall: SEMAR: 2.5 (1.5) vs Standard: 3 (1.5), *p* < .001; celiac artery: 2.5 (1.8) vs 3 (1.5), *p* = .031; SMA: 2.5 (1.4) vs 3 (1.4), *p* = .047; and renal arteries: 3 (1.5) vs 4 (2.3), *p* < .001) (Table 3).

**Fig. 4:**
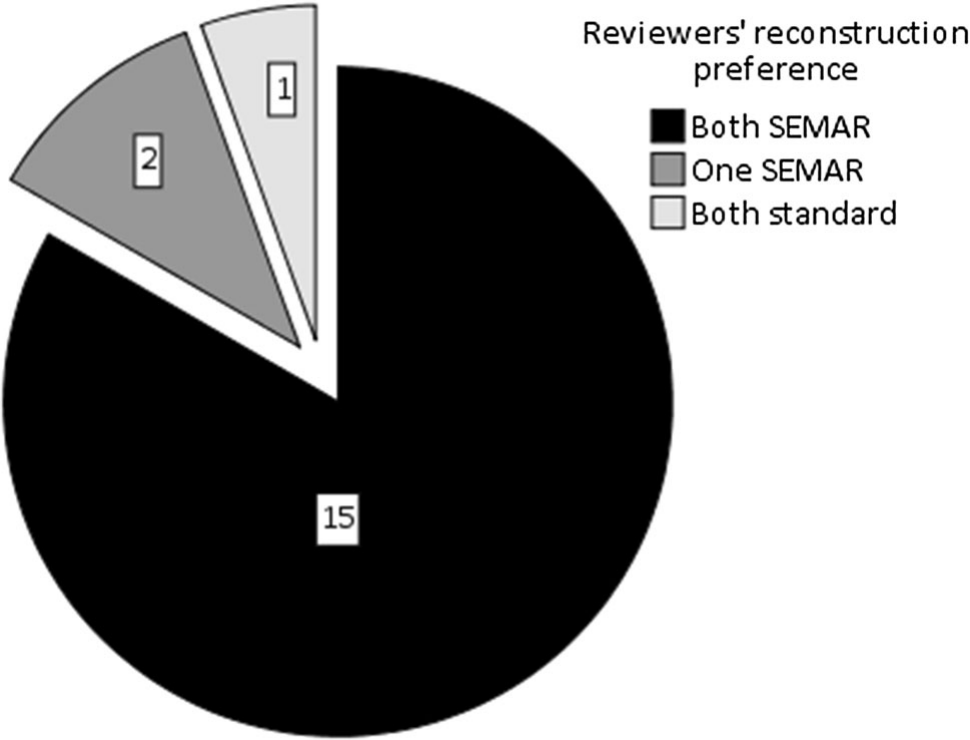
Pie chart showing reviewers’ preference. In 83% (n = 15) of the cases, both reviewers preferred the SEMAR algorithm. Whereas in 11% (n = 2) or 6% (n = 1) of the cases, one of the reviewers or both the reviewers preferred the standard algorithm, respectively

Figure 4 summarizes the reviewers’ preference regarding the CT reconstruction technique. In 83% (*n* = 15) of the cases, both reviewers preferred the SEMAR algorithm.

### Inter-reader Agreement

We found a good inter-reader agreement of *k* = .75 (95% CI .71–.79) over all subjective ratings between both reviewers [17].

## Discussion

Diagnostic confidence of peripheral stent patency in CTA images of patients who have undergone a complex endovascular aortic repair with F/B-EVAR is limited due to metal artifacts. The same problem is well known in coronary stent interventions [18–20]. Currently, the diagnosis of peripheral in-stent stenosis or occlusion in F/B-EVAR patients often depends on secondary signs of (partial) occlusion, e.g., decreased or absence of contrast filling of the peripheral visceral vessel segment, and decreased attenuation or infarction of visceral organs. Early detection of in-stent stenosis is mandatory to prevent total vessel occlusion resulting in renal failure, liver failure, or mesenteric ischemia. We evaluated the image quality of a novel metal artifact reduction algorithm that can be used in a single-energy mode that does not complicate the CT acquisition protocol and does not increase radiation dose [12].

In our study, we found improved image quality with reduced noise, improved CNR, less artifacts, and an improved visualization of stent patency on the CT images after SEMAR reconstruction in all target visceral arteries. Our findings are in agreement with prior studies on metal implants and embolization material, showing that SEMAR is effective in decreasing metal artifacts in single-energy CT images [8, 9, 13–15].

**Fig. 5:**
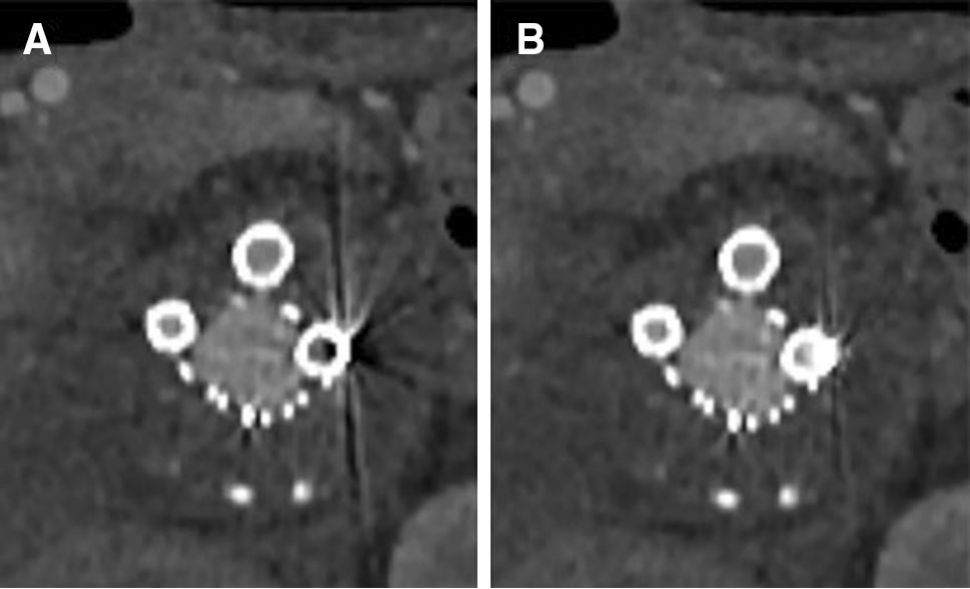
CT images (**A** standard (AIDR 3DE), **B** SEMAR) of a branch connected to the superior mesenteric artery and two branches connected to the renal arteries in a 66-year-old male. Notice the decrease in streaking artifacts in the left renal artery. Although artifacts are reduced significantly, the visualization of stent patency is still limited

We focused on the improvement of artifact reduction and stent patency visualization (Figs. 1, 2). While SEMAR significantly improved the overall image quality in our study, we observed a slight increase in metal density in some image regions (Fig. 5). Although the novel artifacts were easily recognized it did hamper the visualization of stent patency. This phenomenon is most likely due to the algorithm. Similar observations have been recorded in a phantom study of a MAR-algorithm in Gemstone Spectrum Imaging DECT images: Dabirrahmani et al. [21] showed that metal was either under- or overestimated in a phantom model depending on the type of metal.

Overall preference of both reviewers was in favor of the CT images with SEMAR reconstruction in 15/18 (83%) of the cases: in three cases one or both reviewers preferred the standard reconstruction over the SEMAR reconstruction. No difference in amount of stents or stent diameter size was observed in this subsample.

The reconstruction time of the SEMAR reconstruction in F/B-EVAR patients was on average 30 min. This may be a drawback as new CT scans cannot be reconstructed until the SEMAR reconstruction is completed. Possible solutions could be a separate workstation for image reconstruction or to reconstruct the images later. Further refinements are needed to make the reconstruction process faster and more practical.

The forced choice design of the preference analysis could have introduced a bias as the reviewers were forced to choose between the left and the right scans although there might not have been significant differences in the subjective viewing, meaning that the SEMAR algorithm did not improve image quality in these cases but also did not decrease image quality. Despite blinding the reviewers for the image reconstruction, the type of image reconstruction might have been obvious to the reviewers and could potentially have introduced a bias.

The major limitation of our study is the relatively small sample size in a single-center study: we analyzed a total of 35 branches and 26 fenestrations in 18 patients. The results may differ in branches and fenestrations and may depend on the type of peripheral stents; however, due to sample size, we did not perform subanalyses.

**Fig. 6:**
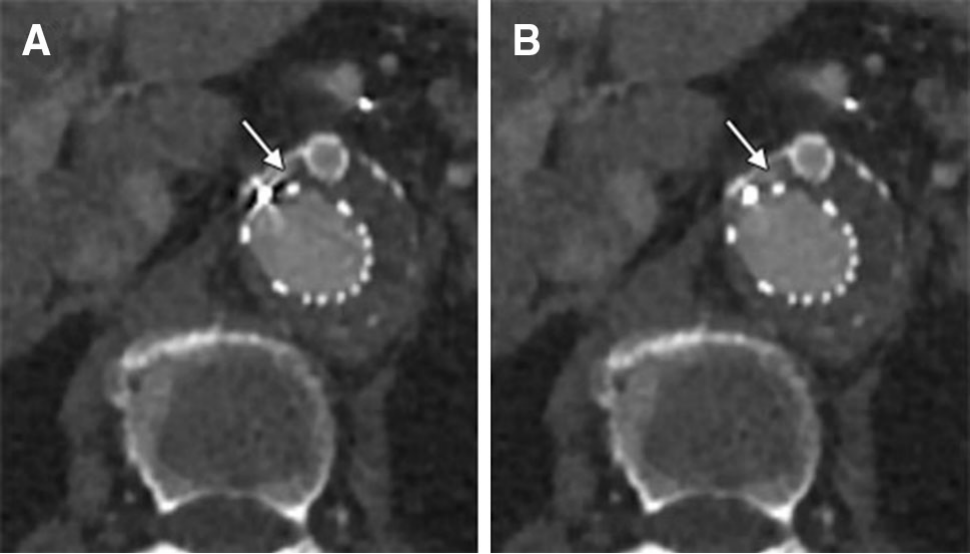
CT images (**A** standard (AIDR 3DE, **B** SEMAR) of a type 1 endoleak in a 70-year-old female. A small amount of contrast is seen immediately ventral of the right renal artery fenestration. The endoleak is visible on both the standard and SEMAR image, but is better appreciated on the SEMAR image (arrow)

In our study, we focused on CT image quality. Due to the fact that the included patients had predominantly early follow-up CT examinations (within 30 days after treatment), and follow-up CT examinations in the same patient were excluded, it was impossible to draw conclusions on in-stent stenoses, occlusion, or endoleaks and are therefore not mentioned in the manuscript. Based on our patient population, we did not find more endoleaks, but small endoleaks near the endograft seemed to be better appreciated (Fig. 6). Whether the improved image quality of the CT examination with SEMAR reconstruction will impact the clinical consequences needs to be addressed in future research.

In conclusion, CTA image quality is improved significantly in reconstructions with the SEMAR algorithm in patients after F/B-EVAR. Further research is warranted to determine the clinical benefit of the SEMAR algorithm in the surveillance of these patients.
